# Luminescence encoding of polymer microbeads with organic dyes and semiconductor quantum dots during polymerization

**DOI:** 10.1038/s41598-022-16065-x

**Published:** 2022-07-14

**Authors:** Lena Scholtz, J. Gerrit Eckert, Toufiq Elahi, Franziska Lübkemann, Oskar Hübner, Nadja C. Bigall, Ute Resch-Genger

**Affiliations:** 1 Division 1.2 Biophotonics, Federal Institute for Materials Research and Testing (BAM), Richard-Willstätter-Str. 11, 12489 Berlin, Germany; 2 Institute for Chemistry and Biochemistry, Free University Berlin, Takustr. 3, 14195 Berlin, Germany; 3 Institute of Physical Chemistry and Electrochemistry, Leibniz Universität Hannover, Callinstraße 3A, 30167 Hannover, Germany; 4 Cluster of Excellence PhoenixD (Photonics, Optics, and Engineering-Innovation Across Disciplines), 30167 Hannover, Germany

**Keywords:** Nanoscience and technology, Optics and photonics

## Abstract

Luminescence-encoded microbeads are important tools for many applications in the life and material sciences that utilize luminescence detection as well as multiplexing and barcoding strategies. The preparation of such beads often involves the staining of premanufactured beads with molecular luminophores using simple swelling procedures or surface functionalization with layer-by-layer (LbL) techniques. Alternatively, these luminophores are sterically incorporated during the polymerization reaction yielding the polymer beads. The favorable optical properties of semiconductor quantum dots (QDs), which present broadly excitable, size-tunable, narrow emission bands and low photobleaching sensitivity, triggered the preparation of beads stained with QDs. However, the colloidal nature and the surface chemistry of these QDs, which largely controls their luminescence properties, introduce new challenges to bead encoding that have been barely systematically assessed. To establish a straightforward approach for the bead encoding with QDs with minimized loss in luminescence, we systematically assessed the incorporation of oleic acid/oleylamine-stabilized CdSe/CdS-core/shell-QDs into 0.5–2.5 µm-sized polystyrene (PS) microspheres by a simple dispersion polymerization synthesis that was first optimized with the organic dye Nile Red. Parameters addressed for the preparation of luminophore-encoded beads include the use of a polymer-compatible ligand such as benzyldimethyloctadecylammonium chloride (OBDAC) for the QDs, and crosslinking to prevent luminophore leakage. The physico-chemical and optical properties of the resulting beads were investigated with electron microscopy, dynamic light scattering, optical spectroscopy, and fluorescence microscopy. Particle size distribution, fluorescence quantum yield of the encapsulated QDs, and QD leaking stability were used as measures for bead quality. The derived optimized bead encoding procedure enables the reproducible preparation of bright PS microbeads encoded with organic dyes as well as with CdSe/CdS-QDs. Although these beads show a reduced photoluminescence quantum yield compared to the initially very strongly luminescent QDs, with values of about 35%, their photoluminescence quantum yield is nevertheless still moderate.

## Introduction

Luminescent polymer beads, encoded either with molecular or nanoscale luminophores, have been increasingly employed in the life and material sciences in the last decades in conjunction with fluorescence spectroscopy, microfluorometry, fluorescence microscopy, and flow cytometry. Such particles are often equipped with surface functional groups to which recognition moieties like proteins and antibodies or analyte-responsive dyes can be attached^1,2^. This opens up many different applications including (bio)imaging, biomedical assays, and chemical sensing^3–8^. While luminescent polymer nanobeads are often employed for cell labeling and assay platforms, commonly larger microbeads are used for bead-based bioassays and spectral multiplexing schemes^8–21^, utilizing either color encoding or recently also lifetime encoding^22^, in conjunction with flow cytometry or fluorescence microscopy. Here, the luminescence color or lifetime of the encoded carrier beads is utilized as an identifying code for the bead surface chemistry and the subsequently bead-bound captured target is then quantified with the aid of an additional spectrally distinguishable fluorescent label. Nano- and micrometer-sized encoded beads can both also be utilized for security, anti-counterfeiting, and authentication applications and printed codes^23–25^.

A common approach to the luminescence encoding of polymer beads presents the swelling of premanufactured polystyrene (PS) or polymethylmethacrylate (PMMA) beads by addition of an apolar organic solvent containing luminophores, which allows the luminophores to permeate the bead matrix^26–28^. Such procedures have been used, e.g., for the fabrication of beads bearing different surface functionalities, which are applied as carriers for bead-based platforms^5^. Alternatively, layer-by-layer coating of premanufactured beads can be performed. This versatile approach involves the step-by-step deposition of layers of oppositely charged polyelectrolytes containing nanocrystals such as colloidal semiconductor quantum dots (QDs) or organic dyes^29–32^. Thereby, only surface staining is achieved and the accordingly altered bead surface chemistry can impose challenges on subsequent bioconjugation steps. Another method is the incorporation of the luminophore during the polymerization reaction, both for organic dyes^33–36^ and different nanocrystals^13,37–43^. Here, the luminescent compound is dissolved or dispersed in the monomer solution or added to the reaction mixture. This procedure can provide a homogeneous luminophore distribution within the beads but requires sufficiently stable emitters with a suitable solubility or dispersibility that can survive the occasionally harsh polymerization conditions^44^.

While procedures for the incorporation of organic dye molecules into polymer beads of different chemical composition, size, and surface chemistry are relatively well established, bead encoding with nanoscale luminophores like QDs with their very broad absorption spectra, narrow emission bands, and high photostability is far more complex^3,17,45^. These luminescent nanocrystals with sizes < 10 nm commonly have a core/shell particle architecture consisting of an inorganic core, an inorganic surface passivation shell, and a stabilizing organic ligand shell, which ensures dispersibility and colloidal stability^46^. As the surface chemistry of the QDs is not only very important for their colloidal stability but also largely controls their photoluminescence (PL) properties, particularly their photoluminescence quantum yield, this introduces considerable challenges for the encoding of polymer beads without risking QD aggregation and a significant loss in QD luminescence^4,38,41,47–49^. This is related to the fact that the bead incorporation process can modify the number of ligands on the QD surface, thereby introducing additional trap states by ligand removal, or require a ligand exchange first to ensure the compatibility of the QD surface chemistry with the monomer/polymer phase, that can also result in luminescence quenching^47^.

The reproducible encoding with nanocrystals like QDs calls for the careful consideration of all parameters controlling their colloidal stability and functionality and hence commonly an adaptation of the encoding procedures established for molecular luminophores. Utilization of a swelling procedure, which has been reported for different types of polymer beads^17,20,47,50^, requires careful control of the pore size distribution within the beads and can lead to inhomogeneous bead staining, a low QD loading density, lack of reproducibility, and subsequent QD leakage^47^. In addition, the swelling procedure and solvents can considerably affect the luminescence properties of the QDs and result in luminescence quenching. Layer-by-layer coating of premanufactured particles of different chemical composition for QDs is time-consuming, can lead to a broadening of the initial bead size distribution, and requires different purification steps resulting in material loss^51–55^. Also, the influence of this encoding procedure on the PL properties of the incorporated QDs has not been yet systematically assessed. The most frequently used procedure for the fabrication of QD-encoded beads presents the incorporation of the QDs during the polymerization reaction, e.g., for the preparation of PS microparticles, or PS beads containing divinylbenzene (DVB) or PMMA^4,13,37–43,48,56–60^ and polyisoprene as well as poly(lactic-*co*-glycolic acid) beads^2,3,45,61^. Employed polymerization techniques include (mini)emulsion and suspension polymerization^4,13,37–43,47,56,58,59^ as well as more complicated microfluidic approaches^9,10,14,16,62^. Nearly all of these procedures utilize Cd-based QDs such as CdSe, CdSe/ZnS or CdTe, which are commonly stabilized with a combination of trioctylphosphine (TOP)/trioctylphosphine oxide (TOPO) ligands. Occasionally, a polymerizable ligand was introduced to the QD surface for better compatibility of the QDs with the reaction mixture and improved bead incorporation^48,57^.

Despite many reports on QD-encoded polymer beads, up to now, the influence of bead incorporation on the PL properties of QDs has not been systematically studied and commonly the PL features of the initial QDs are not or only very roughly compared with those of the resulting QD-stained beads. Particularly, changes in the photoluminescence (PL) quantum yield (PLQY) have not been thoroughly examined, although this property, which equals the number of emitted per number of absorbed photons, largely determines the intensity of the PL signal and bead brightness. Moreover, PLQY provides a direct measure for the quality and tightness of QD surface passivation shells and enables insights into changes in the ligand shell^49,63,64^. This encouraged us to develop a simple procedure to synthesize luminescent PS microbeads with sizes of about 1 μm encoded with hydrophobic organic dyes and ligand-stabilized core/shell QDs via a dispersion polymerization of styrene with minimized loss in initial luminescence. Therefore, representatively for Nile Red (NR) and oleic acid (OA)/oleylamine (OLA)-stabilized CdSe/CdS-core/shell-QDs, different polymerization conditions were systematically examined such as temperature, stirring speed, amount of initiator, reaction time, and the addition of the crosslinker divinylbenzene (DVB) as well as QD surface chemistry and the use of a polymer-compatible QD surface ligand. The characterization of the encoded microbeads comprised the determination of the particle size and size distribution with dynamic light scattering (DLS) and electron microscopy as well as elemental mapping of the QD-encoded beads by energy-dispersive X-ray spectroscopy (EDXS) for QD localization within the beads. In addition, the fluorescence properties of the luminophores before the polymerization reaction and in the beads were determined by PL and integrating sphere spectroscopy as well as confocal laser scanning microscopy (CLSM) in the case of the luminophore-stained beads. Thereby, we could identify parameters that provide strongly luminescent PS microbeads with a narrow size distribution and a minimized, albeit still noticeable loss in PLQY of the bead-incorporated QDs.

## Materials and methods

### Materials

Styrene (≥ 99.0%), divinylbenzene (DVB, 80%), azobisisobutyronitrile (AIBN, 98%), tin(II) 2-ethylhexanoate (92.5–100%), ε-caprolactone (97%), poly(ethylene glycol) (PEG, M_W_ 2,500), trioctylphosphine oxide (TOPO, 99%), 1-octadecene (ODE, 90%), toluene (≥ 99.7%), methanol (≥ 99.8%), isopropanol (≥ 99.8%), oleylamine (OLA, 70–80%), 1-octanethiol (98.5%) and acetone (≥ 99.5%) were obtained from Sigma Aldrich Co. Polyvinylpyrrolidone (PVP, M_W_ 40,000), cadmium oxide (CdO, 99.998%), selenium powder (200 mesh, 99.999%) and oleic acid (OA, 90%) were purchased from Alfa Aesar. Toluene, ethanol and *n*-heptane (all spectr. grade) as well as *n*-hexane (≥ 99%) were obtained from Merck KGaA. Ethanol (abs., 99.9%) and dichloromethane (HPLC grade) were obtained from Chemsolute. Benzyldimethyloctadecylammonium chloride (OBDAC, 98.9%) was purchased from HPC Standards GmbH, Nile red (NR) from Fluka Analytical, *n*-octadecylphosphonic acid (ODPA, > 99%) from PCI Synthesis and tri-n-octylphosphine (TOP, 99.7%) as well as deuterated chloroform (99.8 atom%) from ABCR. All solvents used for the optical measurements were of spectroscopic grade and all chemicals were employed as received without further purification.

### Synthesis of CdSe/CdS quantum dots

The oleic acid (OA)/oleylamine (OLA)-stabilized CdSe/CdS-core/shell-QDs were synthesized according to a modified synthesis described by Carbone et al*.*, Nightingale et al*.* and Chen et al.^65–67^ which is described in detail in the Supplementary Information (SI).

### Synthesis of polyethylene glycol-block-poly(ε-caprolactone)

The *block*-copolymer polyethylene glycol-*block*-poly(ε-caprolactone) (PEG-*b*-PCL) was synthesized according to an adapted procedure by Meier et al.^68^ and is described in detail in the SI.

### Coating of QDs with OBDAC

For the coating of the OA/OLA-stabilized QDs, a spatula tip of OBDAC was added to 100 µL of the QD solution in toluene in a vial. Then, ethanol was added to reach a volume of 1 mL and the mixture was placed on a shaker at 200 rpm for 5 min. The precipitated QDs were then centrifuged with an Eppendorf Microcentrifuge 5415 D at 8000 rpm for 5 min and washed one time with ethanol. The OBDAC-coated QDs were redispersed in 1 mL styrene, sealed, and stored in the refrigerator until further use.

### Synthesis of crosslinked, dye- or QD-encoded polystyrene microbeads

The synthesis of the encoded PS microbeads was performed according to a modified procedure described by Acter et al.^69^ and the crosslinking of the beads was implemented following a procedure from Li et al*.*^70^. First, 36.6 mg of PEG-*b*-PCL were dissolved in 403 µL toluene. The mixture was placed on a shaker at 200 rpm for 30 min to dissolve the copolymer. In the meantime, 1.465 g PVP and 36.6 mg AIBN were sequentially dissolved in 40 mL of ethanol.

For the dye encoding of the polymer beads, 4 mg of Nile Red were dissolved in 4 mL styrene and 200 µL DVB and the mixture was then briefly sonicated. The ethanolic mixture, the dye-monomer mixture, and PEG-*b*-PCL dissolved in toluene were added to a 100 mL two-neck round-bottom flask in this order. Typically, the flask was sealed under argon and heated to 70 °C in an oil bath. The reaction was stirred at 70 rpm for 24 h before cooling to RT. In case any parameter was varied for the bead synthesis, this is explicitly stated in the following section. The resulting particle dispersion was centrifuged with 2000 rcf for 10 min, the supernatant was discarded, and the remaining particles were washed once with ethanol at 1600 rcf for 10 min. For these washing steps, a Multifuge X1R from Thermo Fisher Scientific Inc. was used. The polymer microbeads were then redispersed in ethanol and stored at room temperature in the dark. For the analytical characterization, the particle stock solution was washed additionally three times with ethanol and each time centrifuged for 10 min at 700 rcf to separate smaller beads and remove any remaining synthesis residuals like styrene or AIBN. These washing steps were performed with an Eppendorf Microcentrifuge 5415 D.

For the QD encoding of the polymer beads, 1 mL of a dispersion of OBDAC-coated, oleic acid/oleylamine-stabilized CdSe/CdS-QDs in styrene, prepared as described in the previous section, were added to 3 mL styrene. For the crosslinking of the beads, 50–200 µL DVB were added and the mixture was briefly sonicated. The ethanolic mixture as well as the PEG-*b*-PCL solution in toluene were prepared as described above and added to the flask together with the QD-monomer mixture. The reaction procedure was otherwise performed under the same conditions as employed for the preparation of the dye-encoded microparticles.

### Nuclear magnetic resonance (NMR)

A solution ^1^H-NMR spectrum of the synthesized PEG-*b*-PCL was recorded at RT with a 400 MHz JEOL JNM-ECX400 spectrometer at Free University Berlin. The PEG-*b*-PCL sample was prepared by dissolving 6 mg PEG-*b*-PCL in 700 µL CDCl_3_. The corresponding spectrum, confirming the chemical identity of the copolymer, is displayed in the SI (Fig. S1).

^1^H-NMR (CDCl_3_, 400 MHz): δ = 1.38 (m, 2H, γ), 1.63 (m, 4H, β & δ), 2.30 (m, 2H, α), 3.64 (s, 4H, a & b), 4.05 (t, 2H, ε), 4.21 (t, 2H, b).

The number-average molecular weight *M*_n_ of the synthesized PEG-*b*-PCL was calculated from the ratio of protons of the PEG and PCL signals according to Meier et al.^68^ to be roughly 4840 g/mol.

### Dynamic light scattering (DLS) and zeta potential measurements

DLS and zeta potential measurements of the different microparticles were carried out with a Zetasizer Nano ZS from Malvern Panalytical Ltd. at T = 25 °C in disposable folded capillary cells (DTS1070), also from Malvern Panalytical Ltd. All particles were dispersed in Milli-Q water (Millipore) for these measurements.

### High-angle annular dark-field scanning transmission electron microscopy (HAADF-STEM)

HAADF-STEM measurements were performed using a 200 kV JEOL JEM-2100F-UHR operated at 200 kV and equipped with a field emission gun as well as an Oxford Instruments INCA 200 for energy-dispersive X-ray spectroscopy (EDXS) enabling elemental mapping. The samples were prepared on carbon-coated copper grids (Quantifoil) via drop-casting of the ethanolic microbead dispersion.

### Scanning electron microscopy (SEM)

Scanning electron micrographs were captured with a JEOL JSM-6700F. The samples taken from the ethanolic microbead dispersions were drop-casted on brass holders. The measurements were performed using low acceleration voltage and current (1 kV and 2 µA).

### Atomic absorption spectroscopy (AAS)

AAS measurements were carried out with an AA140 instrument from Varian Inc. with an oxygen/acetylene flame atomizer to determine the Cd(II) concentration in the QD dispersion. Samples of the QD dispersions were prepared by dissolution of the particles with *aqua regia*. Six standard solutions with different Cd(II) concentrations were used to obtain a calibration curve for the quantification of the Cd(II) concentration.

### Absorption spectroscopy

Absorption spectra of the CdSe/CdS-QDs and Nile Red in toluene/styrene and the respective encoded microbeads in ethanol were recorded with a Specord 210plus spectrophotometer from Analytik Jena at RT in (10 × 10) mm quartz glass cuvettes from Hellma GmbH. The different QD and dye-encoded beads for the leaking experiments were dispersed in MilliQ water and measured using the same conditions and instrument settings.

### Fluorescence spectroscopy

Emission spectra of the CdSe/CdS-QDs and Nile Red in toluene/styrene and the respective encoded microbeads in ethanol were recorded with a FSP920 fluorescence spectrometer from Edinburgh Instruments Ltd. at RT in (10 × 10) mm quartz glass cuvettes from Hellma GmbH. The excitation wavelength was set at 350 nm.

### Integrating sphere spectroscopy

The photoluminescence quantum yields (PLQY) of solutions of the luminophores in styrene/toluene and the luminophore-encoded microparticles in ethanol were determined absolutely with a stand-alone Quantaurus integrating sphere setup from Hamamatsu Photonics K.K. The measurements were performed at 25 °C in (10 × 10) mm, long-neck quartz glass cuvettes from Hamamatsu Photonics K. K with an excitation wavelength of 350 nm. For PLQY measurements of transparent luminophore solutions, the respective solvent was used as a blank. For all encoded microbead dispersions, dispersions of unstained plain microparticles of similar size and bead concentration were employed as blank.

### Confocal laser scanning microscopy (CLSM)

CLSM measurements were performed using the confocal laser scanning microscope Olympus FV1000 (Olympus, Germany) based on the motorized inverted microscope Olympus IX81 (Olympus, Germany) and a 60 × water immersion objective (A_N _= 1.2). Transmission images were measured at 488 nm. For the recording of the fluorescence images, for optimal signal intensity, excitation with a 458 nm (87% laser power) and a 355 nm (99% laser power) laser was employed and the fluorescence emission was recorded in the range of 560–660 nm. For these experiments, the microbead stock solutions were washed three times with MilliQ water, redispersed in MilliQ water and diluted to a concentration of 0.2 mg/mL. 1 µL of the sample was applied onto a 0.17 mm glass slide.

### Photostability tests

The short-term photostability of the NR- and QD-encoded beads was examined with the confocal laser scanning microscope Olympus FV1000 from Olympus, Germany based upon the motorized inverted microscope Olympus IX81 from Olympus, Germany, and a 60 × water immersion objective (AN = 1.2). Fluorescence images with a dedicated region of interest (ROI) were recorded with a 458 nm laser for the QD-encoded beads (emission range 580–680 nm) and a 514 nm laser for the NR-encoded beads (emission range 560–660 nm). The image size was set to 512*512 px and a scanning speed of 8 µs/px was employed, resulting in an image acquisition time of 2.1 s. The excitation power was determined to be 1 mW in the beam path for the two excitation light sources. For the sample preparation, the microbead stock solutions were washed three times with MilliQ water, redispersed in MilliQ water and diluted to a concentration of 0.2 mg/mL. 1 µL of the sample were applied onto a 0.17 mm glass slide and left to dry before the measurements.

The long-term stability of the dye NR, the QDs as well as NR- and QD-encoded beads against sunlight was tested with a SUNTEST CPS + setup from Atlas Material Testing Technology GmbH. The dried samples were illuminated for seven days with a power density of 650 W/m^2^ and a maximum chamber temperature of 60 °C. The PL intensity of the dried solid samples of NR, QDs and the NR- and QD-encoded polymer beads was measured in intervals of 24 h using a spectrofluorometer Dual-FL equipped with a Quanta-Phi integrating sphere, both from Horiba Scientific.

## Results and discussion

**Figure 1:**
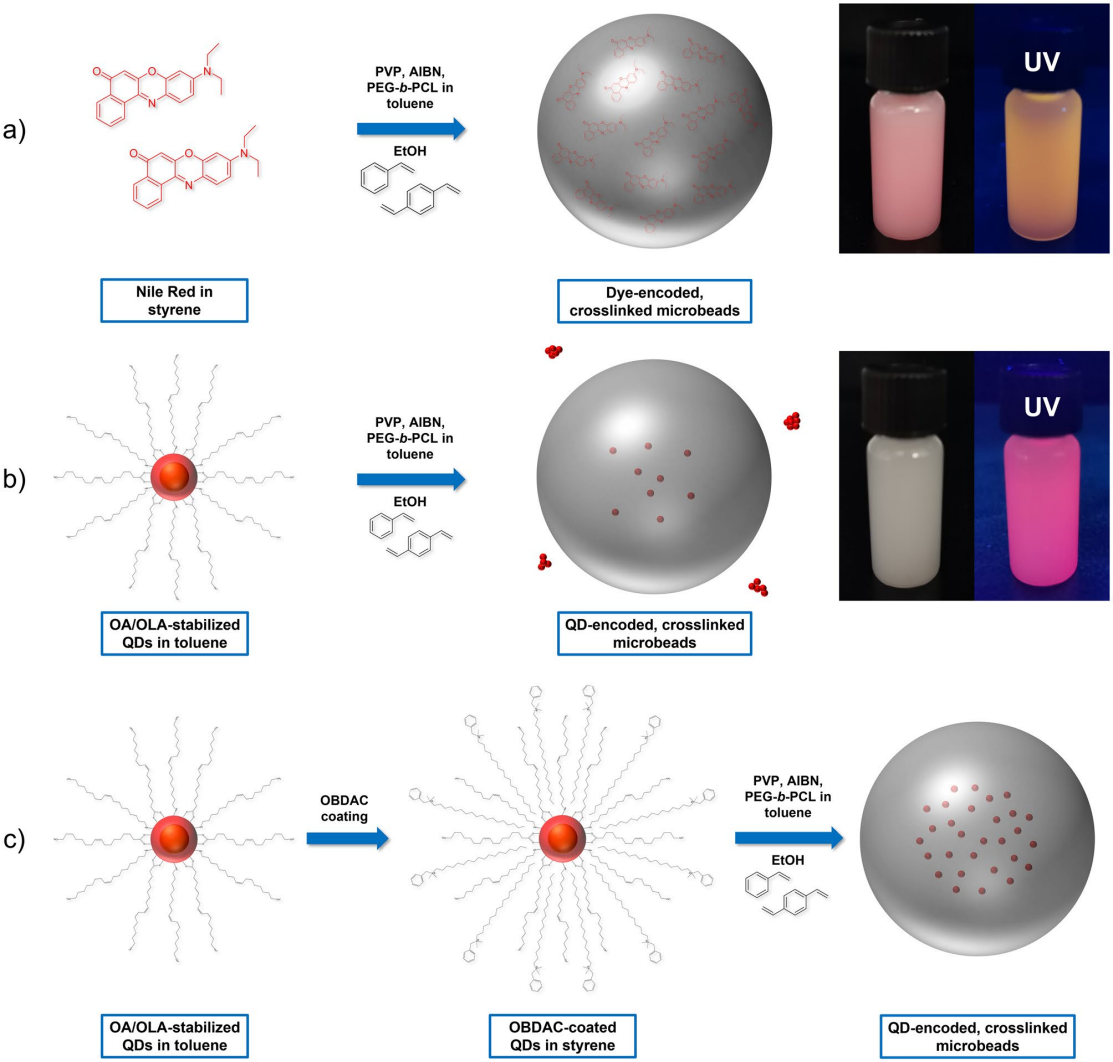
Schematic presentation of the synthesis routes employed for the preparation of the luminescent polystyrene (PS) microbeads, encoded with (**a**) Nile Red, (**b**) OA/OLA-stabilized CdSe/CdS-QDs without pretreatment, and (**c**) OA/OLA-stabilized CdSe/CdS-QDs, synthesized with the developed and optimized procedure, including the pretreatment of the QDs with the polymer-compatible ligand OBDAC. The photographs of the respective ethanolic bead dispersions were taken under day and UV light.

The versatility of dispersion polymerizations, which can be performed in solvents like water or ethanol, in combination with their good reproducibility and high yields render this approach very attractive for the synthesis of luminophore-encoded polymer nanoparticles and microparticles. Polymer nanoparticles with sizes of about 50–200 nm and a narrow size distribution can be synthesized with this classical approach using a monomer, a surfactant, and a radical starter^3,4,35,39,40,43,45,48,56–59,61^. For bead encoding with hydrophobic organic dyes, which are sufficiently stable to survive the polymerization conditions, the luminophore is commonly dissolved directly in the liquid monomer. The synthesis of microparticles with sizes of about 500 nm up to several hundred µm calls for some modifications of this procedure, especially for the incorporation of small nanocrystals like QDs^2,8,10–14,37,38,40–43,47,48,62^. In the following work, the optimization of this procedure is done exemplarily for the solvatochromic, hydrophobic, and sufficiently stable dye Nile Red (NR), which has been utilized by us before to assess and optimize the loading of premanufactured PS nanoparticles and microparticles with a swelling procedure^26,27^. This was done to establish the bead synthesis for the simplest case, a small molecular fluorophore, and show its general suitability for the preparation of homogeneously stained beads. Subsequently, we determined the optimum conditions for the preparation of bright and stable QD-encoded PS microparticles. For this, only one parameter at a time is varied. Thereby, the influence of the copolymer PEG-*b*-PCL, QD coating with OBDAC, and the crosslinking with DVB can be consecutively assessed. The optimized reaction procedure derived for dye and QD encoding of PS microbeads with sizes of 0.5–2.5 μm is schematically displayed in Fig. 1.

### Preparation of polymer microparticles with a narrow size distribution

**Figure 2:**
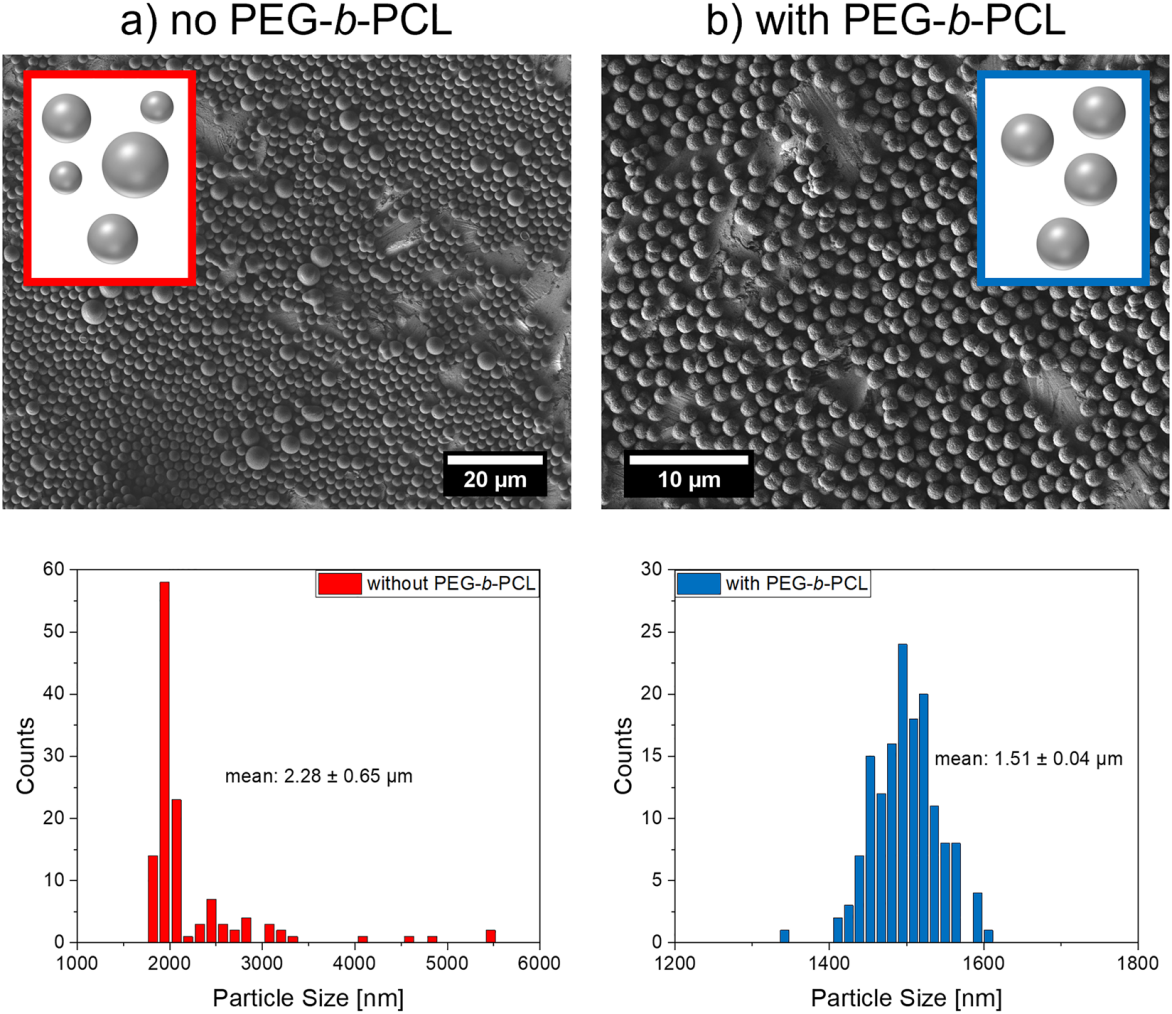
SEM images and particle size distributions of the different PS microbeads prepared: (**a**) without addition of PEG-*b*-PCL (toluene added without PEG-*b*-PCL; synthesis with 500 mg AIBN, no crosslinker) and (**b**) with addition of 36.6 mg PEG-*b*-PCL in toluene (synthesis with 100 mg AIBN, crosslinked with 200 µL DVB). Conditions used for the preparation of both bead types: 75 °C, 70 rpm stirring speed, 24 h reaction time.

PS microbeads prepared by a dispersion polymerization tend to display a broad size distribution. This is mostly caused by the secondary nucleation occurring during the long growth times which leads to differently sized polymer particles. To tackle this challenge, we added the *block-*copolymer PEG-*b*-PCL to the reaction mixture as described by Acter et al.^69^. The addition of this amphiphilic copolymer prevents secondary nucleation and aggregation during bead synthesis by sterically stabilizing the growing particles. The effect of PEG-*b*-PCL on the size distribution of the resulting PS microbeads is visualized in Fig. 2. The SEM images and the derived histograms of the size distribution show a significantly narrower and more regular size distribution with a smaller standard deviation in the presence of PEG-*b*-PCL. While the particles without PEG-b-PCL are generally larger because of the higher amount of AIBN, this influence is still clearly visible. This confirms the beneficial influence of PEG-*b*-PCL on the particle features. Because of these findings, PEG-*b*-PCL was employed in all following syntheses for dye and QD encoded beads to ensure a narrow size distribution of the formed beads.

### Nile Red-encoded microparticles

**Figure 3:**
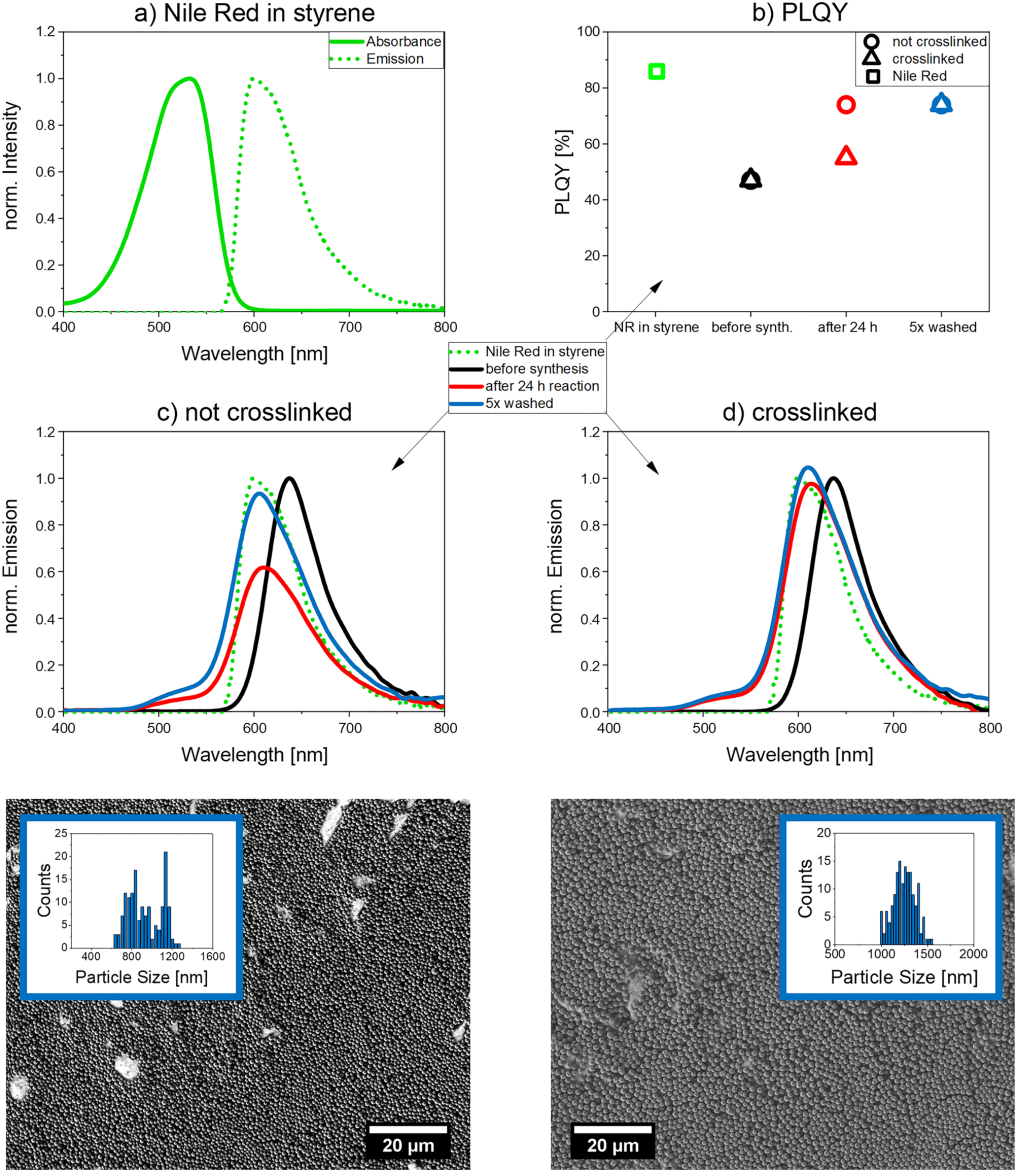
Luminescence properties of NR and NR-encoded PS microbeads. (**a**) Normalized absorbance and emission spectra (λ_exc_ = 350 nm) of NR in styrene; (**b**) PLQY of NR in styrene and in the resulting microbeads in ethanol at different stages; emission spectra (λ_exc_ = 350 nm) of NR-encoded PS microbeads in ethanol prepared (**c**) without and (**d**) with addition of 200 µL of the crosslinker DVB. The emission spectra were normalized to the black emission spectra of the dye NR recorded in the initial reaction mixture prior to polymerization to visualize polymerization-induced changes in fluorescence. For the preparation of the dye-encoded microbeads, the following conditions were used: 65 °C, 100 mg AIBN, 36.6 mg PEG-*b*-PCL, 100 rpm stirring speed, and 24 h reaction time.

The optical properties of NR were determined in styrene before particle synthesis. The corresponding absorption and emission spectra are displayed in Fig. 3. The PLQY of NR in styrene was determined to be 86%.

To evaluate the influence of crosslinking on the PL properties of the beads, NR-encoded microbeads were synthesized without and with the crosslinker DVB using the same amount of dye (1 mg/mL monomer). As derived from SEM images, with a size of 1262 ± 120 nm, the crosslinked beads are larger than the non-crosslinked ones revealing a size of 945 ± 166 nm. The standard deviation of the particle size of both beads is similar, yet slightly higher than for the plain particles without luminophore encoding previously introduced and discussed.

The emission spectra of the polarity probe NR in the initial reaction mixture before polymerization, after 24 h, and after five washing steps are displayed in Fig. 3. The bathochromic shift of the emission band of NR in the polymerization cocktails is ascribed to an increased polarity of the dye environment. Upon polymerization, the NR emission maximum shifts from 637 to 610 nm for both types of microparticles. The PLQY of the reaction mixtures before the synthesis was 47% in both cases. After a reaction time of 24 h, however, PLQY values of 23% and 55% were obtained for the non-crosslinked and crosslinked microparticles. After five washing steps, the PLQY of both bead types amounted to 74%. The decrease in PLQY compared to NR in styrene (86%) is attributed to a slightly increased polarity of the dye environment in the PS beads which is known to reduce the PLQY of NR^26,71^. This is supported by the slight bathochromic shifts of the emission spectra shown in panels c and d of Fig. 3.

### QD-encoded microbeads with fluorescence preservation—influence of QD surface chemistry and crosslinking

Although there are several reports on the preparation of QD-encoded beads, as stated earlier, the influence of the polymerization reaction and the bead matrix on the optical properties of QDs have been only rarely assessed systematically, mostly just by comparison of the emission maxima before and after the reaction^3,4,11–13,35,38–43,48,56,57,60–62^. Up to now, there exist only two examples for PLQY studies of QDs prior to and after bead incorporation, one from Yang et al.^41^ on 3-mercaptopropionic acid-stabilized CdTe in polystyrene and one from Sheng et al.^48^ on TOP/TOPO-stabilized, additionally oligomeric phosphine-coated CdSe/ZnCdS/ZnS in polystyrene. In both cases, a significant loss in PLQY upon QD incorporation into the microbeads was reported^41,48^.

**Figure 4:**
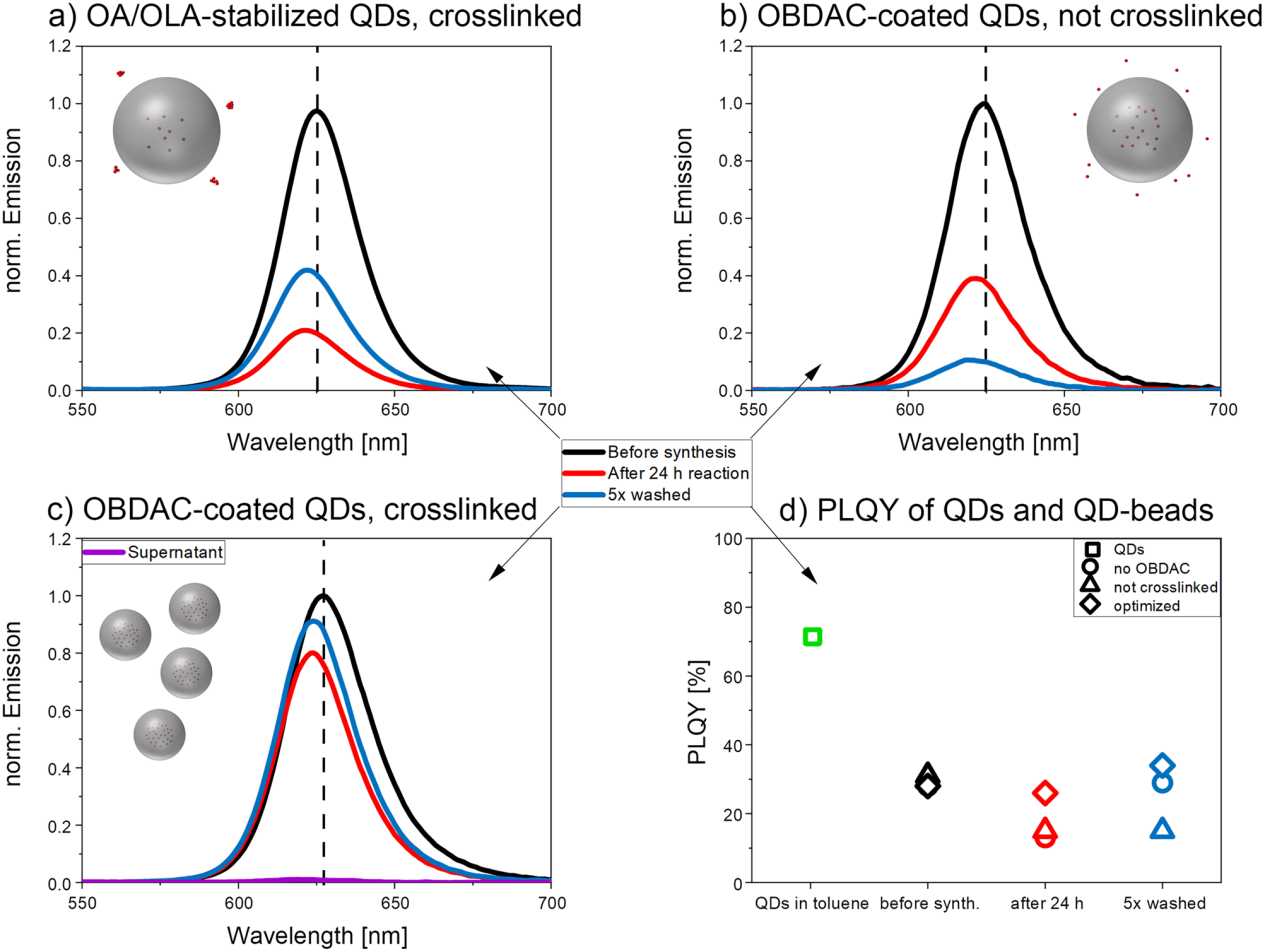
Emission spectra (λ_exc_ = 350 nm) of CdSe/CdS-QDs in the reaction mixture before the polymerization reaction and the resulting QD-encoded PS microbeads in ethanol, obtained for (**a**) OA/OLA-stabilized QDs, crosslinked with DVB, (**b**) OBDAC-coated, OA/OLA-stabilized QDs without crosslinking, with PEG-*b*-PCL, (**c**) for optimized conditions, i.e., OBDAC-coated, OA/OLA-stabilized QDs, addition of PEG-*b*-PCL, and crosslinking with DVB, and (**d**) the corresponding PLQY values obtained at different reaction stages and for the initial QDs. Conditions used for the preparation of the QD-encoded PS beads: 75 °C, 36.6 mg AIBN, 36.6 mg PEG-*b*-PCL, 70 rpm stirring speed, and 24 h reaction time. For the comparison of the PL spectra, the emission intensity of the QDs in the initial reaction mixture was always set to one and the other spectra were scaled accordingly. The observed shift of the emission maxima is attributed to the change in QD environment from initially styrene/ethanol to polystyrene after the polymerization reaction. The increase in PL intensity of the beads in panels (**a**,**c**) washed 5 times with ethanol compared to the PL of the beads after a reaction time of 24 h is attributed to a slight change in bead concentration and bead loss during the washing steps as revealed by the corresponding absorption spectra displayed in the SI (Fig. S4).

**Figure 5:**
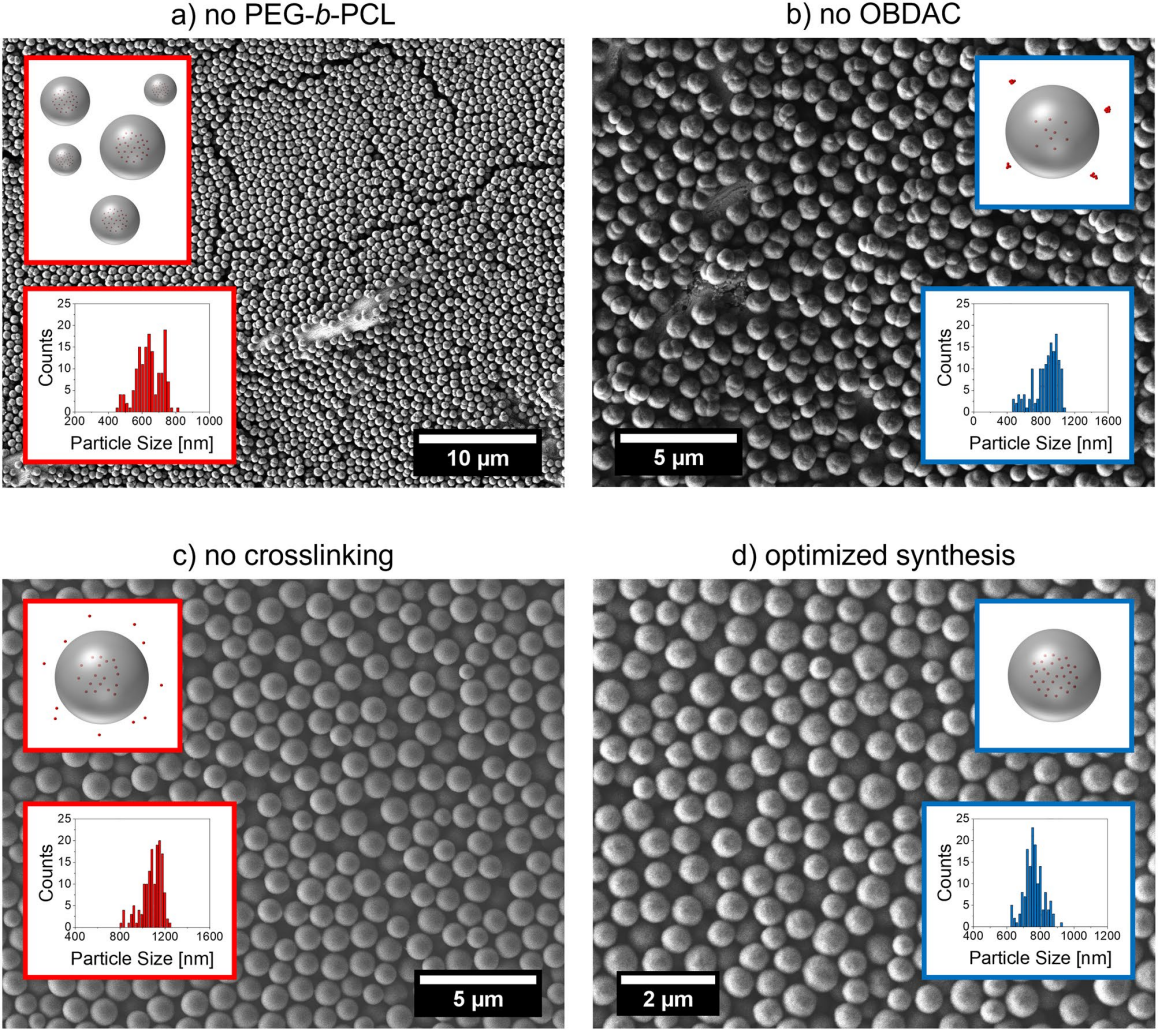
SEM images with the schematic presentation of the CdSe/CdS-encoded microbeads and the size distribution, synthesized from (**a**) OBDAC-coated, OA/OLA-stabilized CdSe/CdS-QDs and styrene (36.6 mg AIBN, toluene added without PEG-*b*-PCL, 200 µL DVB), (**b**) OA/OLA-stabilized CdSe/CdS-QDs and styrene in the presence of the copolymer PEG-*b*-PCL (36.6 mg AIBN, 36.6 mg PEG-*b*-PCL, 200 µL DVB), (**c**) OBDAC-coated, OA/OLA-stabilized CdSe/CdS-QDs and styrene in the presence of PEG-*b*-PCL without the crosslinker DVB (100 mg AIBN, 36.6 mg PEG-*b*-PCL), and (**d**) OBDAC-coated, OA/OLA-stabilized CdSe/CdS-QDs and styrene with the optimized procedure (36.6 mg AIBN, 36.6 mg PEG-*b*-PCL, 200 µL DVB). Reaction conditions for all: 75 °C, 70 rpm stirring speed, 24 h reaction time.

Aiming for bright QD-encoded beads with minimum loss in QD fluorescence and no QD leaking, we assessed and optimized the conditions of the polymerization reaction utilizing DLS, SEM, and PL measurements for bead quality control. The results are summarized in Figs. 4 and 5. Before the synthesis, the structure-analytical and optical properties of the oleic acid (OA)/oleylamine (OLA)-stabilized CdSe/CdS-QDs were examined by TEM as well as absorption and PL spectroscopy and the QD concentration of the dispersion used was determined by AAS (for both see SI, Fig. S2). As a tool for bead quality, we focused here first on the PL properties of the QD-encoded beads with special emphasis on the spectral position and spectral width (FWHM: full width at half maximum) of the QD emission band, which correlate with QD size and size distribution, and PLQY which is independent of bead size and the number of incorporated QDs. The structure-analytical characterization of the different types of QD-encoded beads obtained from QDs with different surface chemistries and without and in the presence of a crosslinker are detailed in the following section, thereby assessing features such as bead size, size distribution, and surface morphology as well as QD distribution within the beads.

#### Bead-encoding with OA/OLA-stabilized CdSe/CdS-QDs

In a first attempt to produce QD-encoded beads, OA/OLA-stabilized CdSe/CdS-QDs dispersed in toluene were precipitated by addition of ethanol, separated by centrifugation (5 min at 8000 rpm with an Eppendorf Microcentrifuge 5415 D), and redispersed in styrene. As depicted in Fig. 4a, the QD emission maximum shifts only slightly from 625 to 622 nm due to the change in QD environment and the FWHM of the PL spectra barely changes for bead incorporated QDs. This indicates that the QD size and size distribution are not altered during the polymerization reaction. The formed QD-encoded beads crosslinked with DVB and dispersed in ethanol show a decrease of 80% compared to the initial intensity before the synthesis and PLQY decreased from 28 to 13% (Fig. 4b). After bead purification by washing with ethanol (5 washing-centrifugation cycles), PLQY increased by about 16% reaching a value of 29%. This is ascribed to the presence of free, possibly damaged QDs after bead preparation, which exhibit a low PLQY or are even dark, i.e., non-emissive and contribute only to the absorption of the dispersion, thereby distorting the resulting PLQY of the bead dispersion. These results indicate that under these conditions, the compatibility of the QD surface chemistry with the polymer matrix is poor, as many QDs are obviously not included in the PS particles formed. Also, the polymerization conditions clearly affect QD fluorescence, possibly by modifying the ligand shell. The PL properties of the synthesized beads (emission maxima, FWHM, PLQY) encoded with QDs as well as those encoded with NR are also summarized in the SI (Table S3).

#### Bead-encoding with OBDAC-coated, OA/OLA-stabilized CdSe/CdS-QDs

To better shield and anchor the QDs in the bead matrix, we examined the influence of an additional, polymer-compatible surface ligand, here OBDAC, on the reaction outcome. OBDAC supposedly intercalates with the initially present OA/OLA ligand shell, acting as an additional organic coating wrapped around the QDs. Thereby, ligand exchange and removal are being avoided which can introduce defect and trap states at the QD surface leading to a reduction in PL intensity and PLQY^72^. A comparison of the PL spectra and PLQY of the OBDAC-coated OA/OLA-stabilized and the uncoated QDs shown in the SI (Fig. S2) reveals the absence of spectral shifts and changes in the spectral width of the QD luminescence band. PLQY drops from 72% observed for the as-prepared, OA/OLA-stabilized CdSe/CdS-QDs in toluene to 68% for the OBDAC-coated QDs by only 4%. As observed for the OA/OLA-stabilized QD, the QD emission maximum shifts from 627 to 624 nm for the microbeads containing OBDAC-coated QDs and FWHM was barely affected, suggesting no change in QD size and size distribution during polymerization (Fig. 4c). As shown in Fig. 4b, the PLQY values of the QDs in the reaction mixture before the start of the polymerization reaction was about 28% for both OA/OLA-stabilized QDs and QDs additionally coated with OBDAC. However, in the latter case, PLQY of the resulting QD-encoded microbeads reached 26% after 24 h for the OBDAC-coated, OA/OLA-stabilized QDs, which exceeds the PLQY value resulting for the former (13%) by a factor of 2. For the washed microbeads encoded with OBDAC-coated, OA/OLA-stabilized QDs, a PLQY of 34% was obtained, which is slightly higher (by 5%) than the PLQY of the microbeads containing OA/OLA-stabilized QDs.

#### Crosslinking of QD-encoded beads

A well-known challenge luminophore-encoded beads have to master is the prevention of luminophore leaking under application relevant conditions like washing steps mandatory for bead purification or the presence of proteins or surfactants like streptavidin often used for bioconjugation reactions. As washing of the QD-encoded PS microparticles with ethanol leads to QD leakage even for the OBDAC-coated, OA/OLA-stabilized QDs indicated by a loss in PL intensity, therefore, up to 5% DVB (referring to the amount of styrene used) was added to the polymerization cocktail as a second monomer to crosslink the PS matrix. Subsequently, we examined the influence of bead crosslinking on QD PL features. The emission spectra of the reaction mixture before the polymerization as well as after a reaction time of 24 h and after five consecutive washing steps with ethanol displayed in Fig. 4d clearly demonstrate the beneficial effect of the crosslinker. As shown in this figure, the QD emission maximum shifts from 627 to 624 nm due to the change in QD environment for OBDAC-coated, OA/OLA-stabilized QDs in the presence of DVB. Particles without DVB suffer from a significant loss in PL intensity during bead formation, inhibiting only about 40% of the initial PL intensity and during the washing steps, it even decreases to about 10% of the initial PL intensity. Crosslinking considerably reduces the diminution in PL intensity and helps to prevent QD leaking during bead purification (see also forthcoming section). The latter is indicated by the minimum PL detectable in the supernatant of the washed, crosslinked QD-encoded PS beads (optimized procedure). As shown in Fig. 4, DVB also affects the PLQY of the bead incorporated QDs. While with values of 31% and 28%, the PLQY of the QDs in the polymerization cocktail were very similar without and with crosslinker, the beneficial influence of DVB became apparent after bead formation. PLQY of the crosslinked beads reached a value of 26% exceeding PLQY of the non-crosslinked ones of 15% by a factor of almost 2. The favorable influence of the crosslinker became even more pronounced after five washing steps with PLQY values of 34% for the crosslinked and 15% for the non-crosslinked beads. Apparently, by tightly encapsulating the QDs during bead formation at an early stage of the reaction, DVB cannot only circumvent QD leaking, yet also prevent damage to the OBDAC-coated, OA/OLA-stabilized CdSe/CdS-QDs and shields them from ethanol, which can induce PL quenching, e.g., by irreversible aggregation of the QDs or removal of surface ligands.

### Structure analytical characterization of QD-encoded beads

#### Bead size

Characterization of the QD-encoded beads obtained with OA/OLA-stabilized and OBDAC-coated, OA/OLA-stabilized QDs under identical reaction conditions (75 °C, 36.6 mg AIBN, 70 rpm stirring speed) using DLS and electron microscopy revealed considerable differences in bead size. While in the presence of the former, the bead size amounted to 872 ± 150 nm, for the latter, a bead size of 768 ± 57 nm is obtained (sizes determined from SEM images). The corresponding SEM images with the bead size distributions are shown in Fig. 5.

A comparison of the SEM images of not crosslinked and crosslinked PS particles displayed in Fig. 5 reveals a slightly rougher surface of the crosslinked beads compared to the smoother bead surface obtained without DVB. Also, the presence of DVB increases the bead size. While the crosslinked beads (prepared with 36.6 mg AIBN) have a size of 768 ± 57 nm, the size of the beads without DVB, yet with 100 mg AIBN, amounts to 1097 ± 91 nm as determined from SEM images. The larger size of the latter is partly attributed to the higher amount of AIBN. Crosslinked particles often tend to be larger than their non-crosslinked counterparts, even for otherwise equal reaction conditions (see Fig. 3 for NR-encoded microbeads). This difference can be attributed to changed reaction dynamics introduced by the crosslinker, e.g., by interfering with the particle nucleation^73^. The size distribution of the optimized, QD-encoded beads is very similar to that of the plain beads with a relatively small standard deviation. However, as the focus of our study was on the preservation of a high PLQY of the encapsulated QDs and the prevention of QD leaking, this increase in bead size was not relevant here. Also, for most applications of such QD-encoded beads, this increase in particle size by about 25% is not important.

### QD distribution within the polymer beads

**Figure 6:**
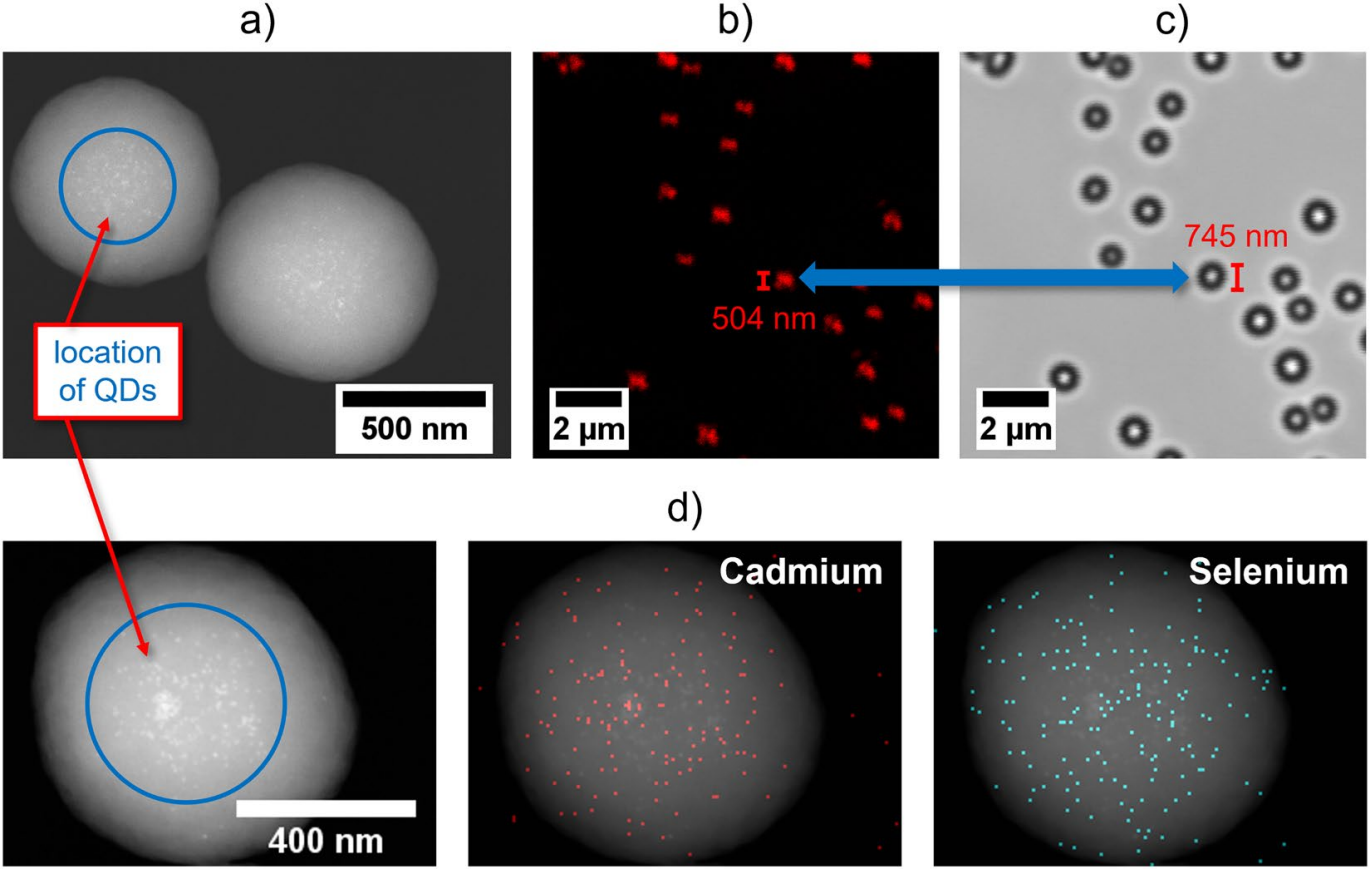
Localization of the QDs inside the PS microbeads, determined (**a**) with a STEM image of DVB-crosslinked PS microbeads encoded with OBDAC-coated, OA/OLA-stabilized CdSe/CdS-QDs, revealing the location of the QDs in the microbead core region; with CLSM images of the same particles measured (**b**) in fluorescence and (**c**) in transmission, including a size comparison of the fluorescent area and the particle diameter derived from the transmission image and (**d**) with EDXS analysis of the same DVB-crosslinked PS microbeads encoded with OBDAC-coated, OA/OLA-stabilized CdSe/CdS-QDs. Reaction conditions of particles in all images: 75 °C, 36.6 mg AIBN, 36.6 mg PEG-*b*-PCL, 70 rpm stirring speed, 24 h reaction time.

The QD distribution within the PS microparticles, which can be relevant, e.g., for all types of applications relying on energy transfer from the encoding fluorophores to surface-bound fluorophores, was exemplarily assessed for selected beads encoded with OBDAC-coated, OA/OLA-stabilized CdSe/CdS-QDs utilizing STEM with EDXS and CLSM. To obtain a detailed insight into the QD distribution in a single microbead, STEM images were taken with an acceleration voltage of 200 kV while the distribution of Cd, Se, and S constituting the core/shell QDs within the microbead was derived from EDXS measurements. The obtained images are displayed in Fig. 6. The STEM image indicates the localization of the QDs in the bead core region. This also reveals only very little agglomeration or aggregation of the single QDs within the bead. The EDXS maps shown in Fig. 6d indicate the presence of Cd and Se in the encoded microbeads, which confirms the successful incorporation of the CdSe/CdS-QDs into the beads. Additional EDXS maps of C and S are included in the SI (Fig. S5). As can be seen in Fig. 6b,c, the CLSM images of the QD-encoded beads support that the origin of PL mainly originates from the bead core. This becomes obvious by comparing the fluorescence with the transmission image, with the latter showing larger particles than the bright areas in the fluorescence image. The QD accumulation in the core region is ascribed to the hydrophobicity of the OBDAC-coated, OA/OLA-stabilized QDs that, together with the crosslinking with DVB, favors QD incorporation in the first bead seeds formed from the start of the nucleation reaction, thereby removing them as far as possible from the ethanolic part of the reaction mixture.

### Stability and leaking studies with encoded beads

To further investigate the stability of dye and QD encoding, the differently prepared and purified encoded beads were incubated in different media (MilliQ water, phosphate-buffered saline solution (PBS), Dulbecco’s Modified Eagle′s Medium (DMEM)) for one hour at 37 °C. Then, the beads were separated with centrifugal filter units (10 kDa, Amicon Ultra, Merck Millipore) and the amount of released dye molecules or QDs in the supernatant was photometrically determined. The results of the leaking studies are displayed in the SI (Fig. S6). These experiments revealed high leaking stability of NR molecules and QDs in the microbeads, as the detected amount of luminescent compound was far below 1% in most cases. For DVB crosslinked microbeads with OBDAC-coated, OA/OLA-stabilized CdSe/CdS-QDs, the amount of free QDs was below 1% for MilliQ water and PBS, and below 2% for DMEM. The same applies to the amount of free dye for DVB crosslinked microbeads with NR. For the NR-encoded beads without crosslinking, less than 1% of free dye could be detected for all three media. For OBDAC-coated, OA/OLA-stabilized CdSe/CdS-QDs, QD release from microbeads prepared without DVB was below 1% in MilliQ water and DMEM, and about 1% for PSB. These results confirm the excellent leaking stability of our NR- and QD-encoded beads.

### Photostability studies of the NR- and QD-encoded beads

The short-term photostability of the NR- and QD-encoded microbeads was assessed with the CLSM. The long-term stability was estimated with a sunlight simulator, here also in comparison to the dye NR and the QDs. The corresponding data are displayed in the SI (see Fig. S7).

The short-term CLSM measurements revealed a roughly exponential decay of the luminescence intensity of the NR-encoded beads and a nearly linear decay for the QD-encoded beads. After 20 scans, corresponding to about 42 s, the NR-encoded beads were left with about 48% of their initial luminescence intensity, while the QD-encoded beads preserved 67%. of their initial luminescence.

As expected, the NR-encoded beads showed a very limited photostability with a significant loss in luminescence even after short intervals of light exposure. The NR-encoded beads, however, still show a luminescence after 24 h of illumination at a power density of 650 W/m^2^ in the sunlight simulator, albeit a much smaller remaining luminescence than the QD-encoded beads. After illumination for 72 h, the NR-encoded beads show a more or less complete loss in luminescence after 72 h while the QD-encoded beads are still luminescent after 120 h of light exposure.

### Optimization of the polymerization conditions and assessing the tunability of bead size

**Figure 7:**
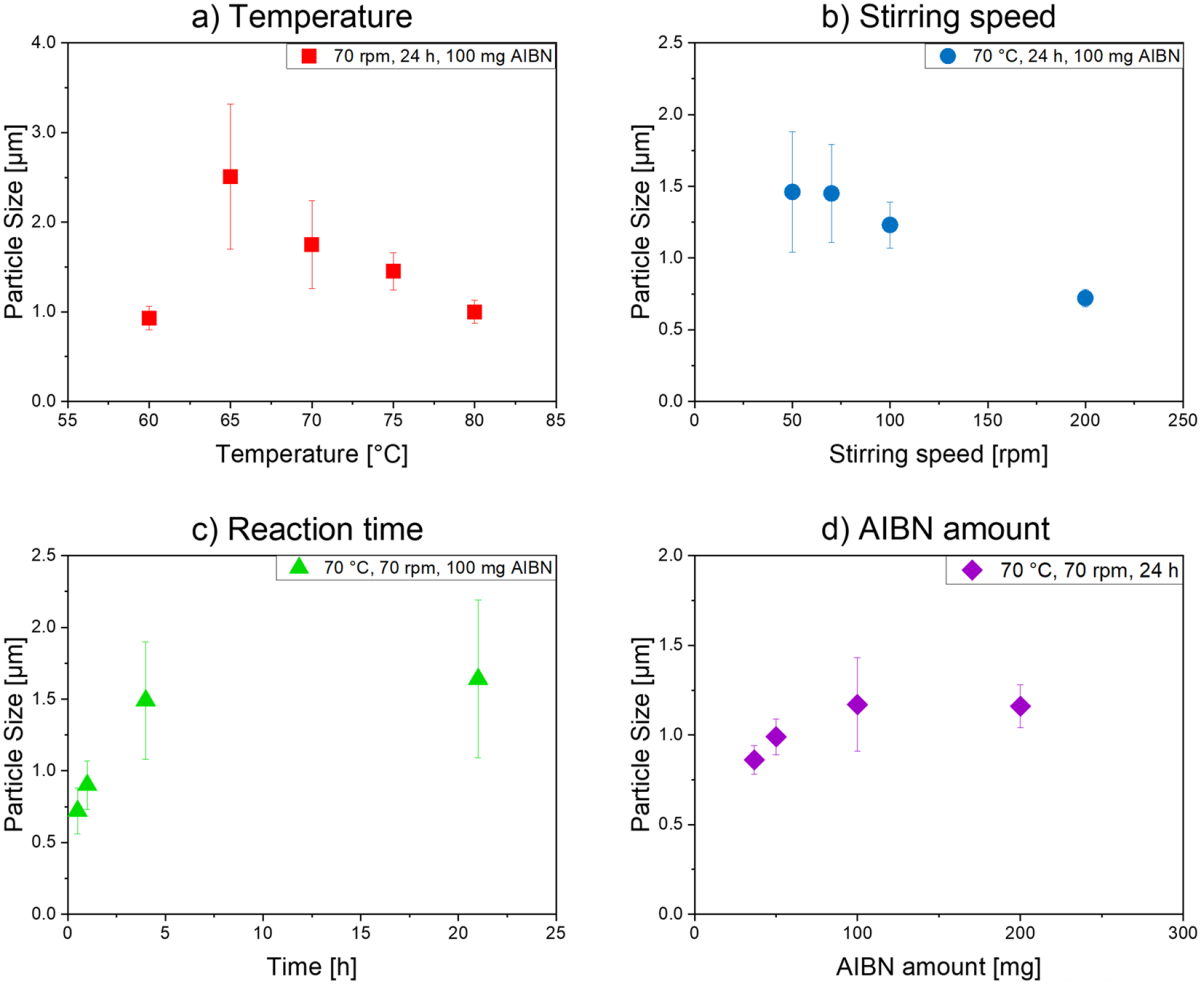
Particle size of DVB-crosslinked PS microbeads encoded with OBDAC-coated, OA/OLA-stabilized CdSe/CdS-QDs, as determined by DLS, obtained as a function of systematically varied (**a**) reaction temperature, (**b**) stirring speed, (**c**) reaction time (samples not purified), and (**d**) amount of AIBN. In all cases, 36.6 mg PEG-*b*-PCL in toluene and the same amount of OBDAC-coated, OA/OLA-stabilized CdSe/CdS-QDs were used.

Subsequently, we performed screening studies of the size tunability of the polymer microbeads encoded with OBDAC-coated, OA/OLA-stabilized CdSe/CdS-QDs utilizing our polymerization procedure to assess its flexibility and identify the most relevant parameters for bead size control. Therefore, different parameters of the previously optimized polymerization reaction were varied, and the size of the resulting beads was determined with DLS. Assessed parameters included temperature (varied between 60 and 80 °C), stirring speed (50–250 rpm), reaction time (0.5–24 h), and AIBN amount (36.6–200 mg), which were modified while keeping other synthesis conditions constant. As shown in Fig. 7, panel a, an important factor with a considerable influence on particle size is the reaction temperature, the increase of which leads to a decrease in bead size. This is attributed to an increased number of seeds formed simultaneously at high temperatures which then automatically results in a smaller particle size. The microbeads formed at a reaction temperature of 60 °C are relatively small and the resulting bead dispersion has a low bead concentration, indicating that the polymerization reaction is significantly slowed down and incomplete at this temperature. As depicted in panel b of Fig. 7, an increase in stirring speed favors the formation of smaller microbeads by decreasing the size of the PVP micelles in which the polymer beads grow. The time dependence of the bead growth behavior shown in Fig. 7c indicates that for reaction times of up to four hours, the particle size increases fast and almost linearly. Then, the growth speed significantly slows down. Also, the amount of the radical initiator AIBN can affect microbead size (Fig. 7d). As to be expected, usage of an increased amount of AIBN, and thus the presence of a larger number of radicals, speeds up the polymerization reaction and provides larger beads. An AIBN amount exceeding 200 mg, however, causes microbead aggregation during the polymerization reaction.

## Conclusion and outlook

In this work, we developed optimized procedures for the encoding of polymer microbeads with hydrophobic, organic dyes and semiconductor quantum dots (QDs) added during the polymerization representatively for Nile Red (NR) and CdSe/CdS QDs stabilized with oleic acid (OA) and oleylamine (OLA). Special emphasis was dedicated to the preservation of the initial photoluminescence (PL) of the QD during bead synthesis and used to optimize the preparation of the QD-encoded beads which has been rarely systematically assessed before. This also provides a deeper insight into how the bead formation reaction and some of its parameters influence QD luminescence. By careful parameter adjustment, the synthesis of luminescent microbeads with narrow size distribution and stable emission properties as well as a relatively high photoluminescence quantum yield (PLQY) could be realized, although the PLQY is still reduced compared to the initial PLQY of the QDs. A minimization of PLQY loss for the bead-incorporated QDs was achieved by using the ligand benzyldimethyloctadecylammonium chloride, that was wrapped around the QDs, making the QD surface chemistry better compatible with the polymer matrix, and the crosslinker divinylbenzene (DVB) to prevent QD leakage.

The developed optimized dispersion polymerization approach is simple and paves the road for the facile usage and combination of different materials such as QDs with varying compositions, dyes or other luminescent nanocrystals as encoding materials for the microbeads which can then be employed for optical multiplexing and the combination with magnetic nanoparticles, e.g., for immuno-separation. By introducing functional groups to the microbead surface during bead synthesis, particles with different surface chemistries can be made enabling further processing steps like bioconjugation reactions. In the future, we plan to expand these studies on the influence of the polymerization reaction and bead incorporation on the luminescence properties of nanocrystals to QDs with systematically varied surface chemistries including the chemical nature and thickness of the surface passivation shell, QD surface chemistry, and morphology. These encoded beads will then be employed for follow-up studies regarding possible applications such as multiplexed bioassays.

## Supplementary Information

Supplementary Information.

## Data Availability

All data generated or analyzed during this study are included in this published article (and its Supplementary Information files) or are available upon request.
