# Supplementary material for: Luminescence encoding of polymer microbeads with organic dyes and semiconductor quantum dots during polymerization
Source: Sci Rep. 2022 Jul 14;12:12061. doi: 10.1038/s41598-022-16065-x (PMC9283474; doi:10.1038/s41598-022-16065-x)
Supplement: Supplementary file 1 — Supplementary Information. [file 41598_2022_16065_MOESM1_ESM.docx]

Supplementary Information for:

**Luminescence Encoding of Polymer Microbeads with Organic Dyes or Semiconductor Quantum Dots during Polymerization**

Lena Scholtz^1,2^, J. Gerrit Eckert^3^, Toufiq Elahi^2^, Franziska Lübkemann^3,4^, Oskar Hübner^1,2^, Nadja C. Bigall^3,4^, Ute Resch-Genger^1,^*

^1^Bundesanstalt für Materialforschung und -prüfung (BAM), Division 1.2 Biophotonics, Richard-Willstätter-Str. 11, 12489 Berlin, Germany.

^2^Institute for Chemistry and Biochemistry, Free University Berlin, Takustr. 3, 14195 Berlin, Germany.

^3^Institute of Physical Chemistry and Electrochemistry, Leibniz Universität Hannover, Callinstraße 3A, 30167 Hannover, Germany.

^4^Cluster of Excellence PhoenixD (Photonics, Optics, and Engineering − Innovation Across Disciplines), 30167 Hannover, Germany.

* ute.resch@bam.de, Phone: +49 (0)30 8104 1134

1. **Synthesis of CdSe/CdS-core/shell-QDs**

The oleic acid (OA)/oleylamine (OLA)-stabilized CdSe/CdS-core/shell-QDs were synthesized according to a modified synthesis described by Carbone *et al.*, Nightingale *et al.* and Chen *et al*.^65-67^

*Synthesis of CdSe cores*. In the first step, wurtzite CdSe cores were synthesised according to Carbone *et al*.^65^ For the synthesis, 120 mg CdO, 560 mg ODPA and 6 g TOPO were degassed for 1 h at 150 °C. The mixture was heated under argon flow at 300 °C and 2 mL TOP were injected. After heating to 380 °C and a retention period of 10 min, 3.6 mL of a previously prepared TOP/Se solution (120 mg/3.6 mL) was swiftly injected. The temperature was allowed to rise to 380 °C again before the reaction was quenched by adding 5 mL ODE and cooled down to 70 °C. At this point, 5 mL toluene was added. The resulting particles were precipitated by methanol/isopropanol (1:2) and redispersed in 2 mL hexane.

*Synthesis of Cd(oleate)_2_*. The Cd(oleate)_2_ precursor solution was synthesised according to Nightingale *et al*.^66^ For the synthesis of a 0.5 M Cd(oleate)_2_ solution, a mixture of 1.284 g CdO, 12.94 mL oleic acid and 7.04 mL ODE were degassed for 10 min at 100 °C. Under argon flow, the dispersion was heated to 180 °C and kept there for 60 min under vigorous stirring. To remove water as a side product, the mixture was cooled to 120 °C and degassed for 45 min. The solution was then used as prepared.

*Synthesis of CdS shells*. The growth of the CdS surface passivation shell was performed according to an adapted synthesis by Chen *et al*.^67^ 100 nmol of the CdSe cores (90–100 µL) were dispersed in 3 mL OLA and ODE, respectively and degassed for 30 min at 90 °C. In the meantime, the S and Cd precursor solutions were prepared. For the desired shell thickness of 5 monolayers, 758 µL Cd(oleate)_2_ and 68 µL 1-octanethiol were diluted to 7 mL with ODE. Under argon flow, the flask was heated in two steps to 310 °C. At 240 °C, the injection of the previously prepared Cd(oleate)_2_ and 1-octanethiol solution via syringe pump (6 mL, 3 mL/h) was started. After two hours, 1 mL oleic acid was injected, and the temperature was maintained at 310 °C for another hour. Finally, the reaction mixture was cooled down to room temperature, and the particles were precipitated by addition of acetone, centrifuged, and redispersed in toluene.

1. **Synthesis and ^1^H NMR of polyethylene glycol-*block*-poly(ε-caprolactone)**

The *block*-copolymer polyethylene glycol-*block*-poly(ε-caprolactone) (PEG-*b*-PCL) was synthesized according to an adapted procedure by Meier *et al*.^68^

In short, 800 mg of poly(ethylene glycol) was placed in a dry flask and 1536 µL of ε-caprolactone was added. The mixture was then stirred in a preheated oil bath at 130 °C for 5 min followed by addition of one drop of Sn(II) 2-ethylhexanoate to initiate and catalyze the reaction. The reaction temperature was kept at 130 °C for 3 h before the reaction mixture was rapidly cooled with the aid of an ice bath leading to the precipitation of a solid product. The solid product was then dissolved in a small amount of dichloromethane and precipitated by addition of *n*-heptane. The recrystallized polymer was filtered and washed several times with *n*-heptane before drying and subsequently characterized by nuclear magnetic resonance spectroscopy (solution ^1^H-NMR) from which the number-averaged molecular weight was calculated.


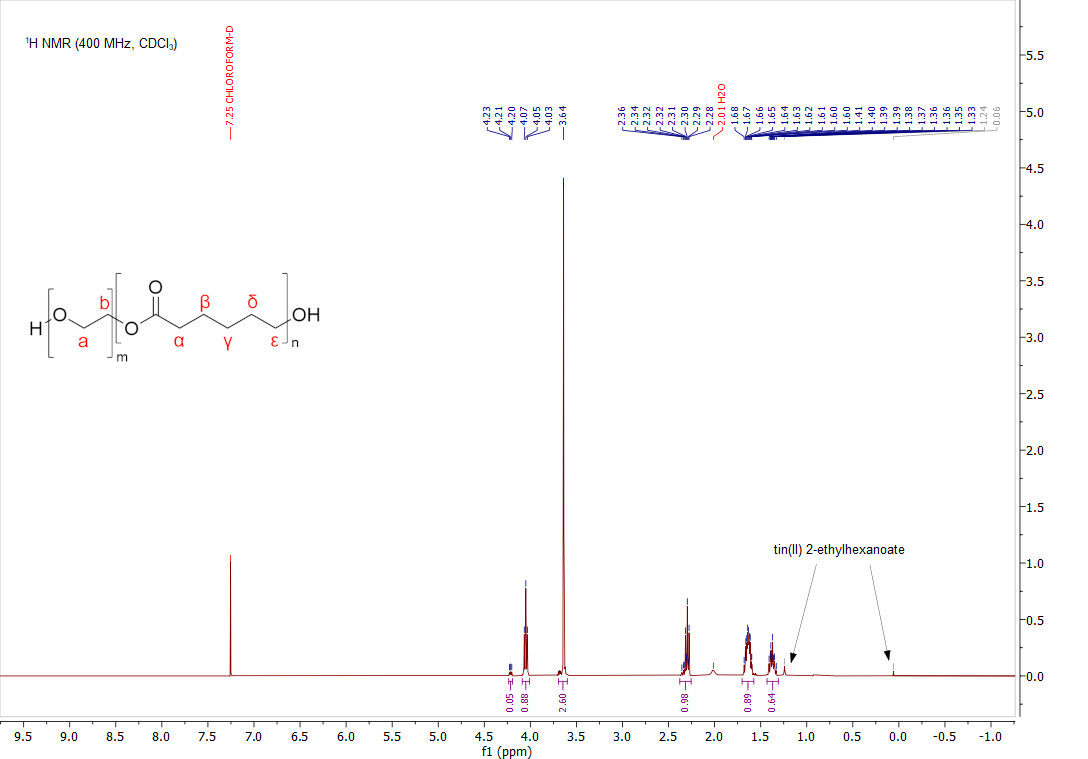


**Figure S1:** 1H NMR spectrum (400 MHz, CDCl_3_) with structural formula of the synthesized polyethylene glycol-block-poly(ε-caprolactone) (PEG-b-PCL) with all compound peaks, two solvent peaks and two impurity peaks that can be attributed to the used catalyst tin(II) 2-ethylhexanoate.

1. **Electron microscopy and optical properties of CdSe/CdS-QDs**


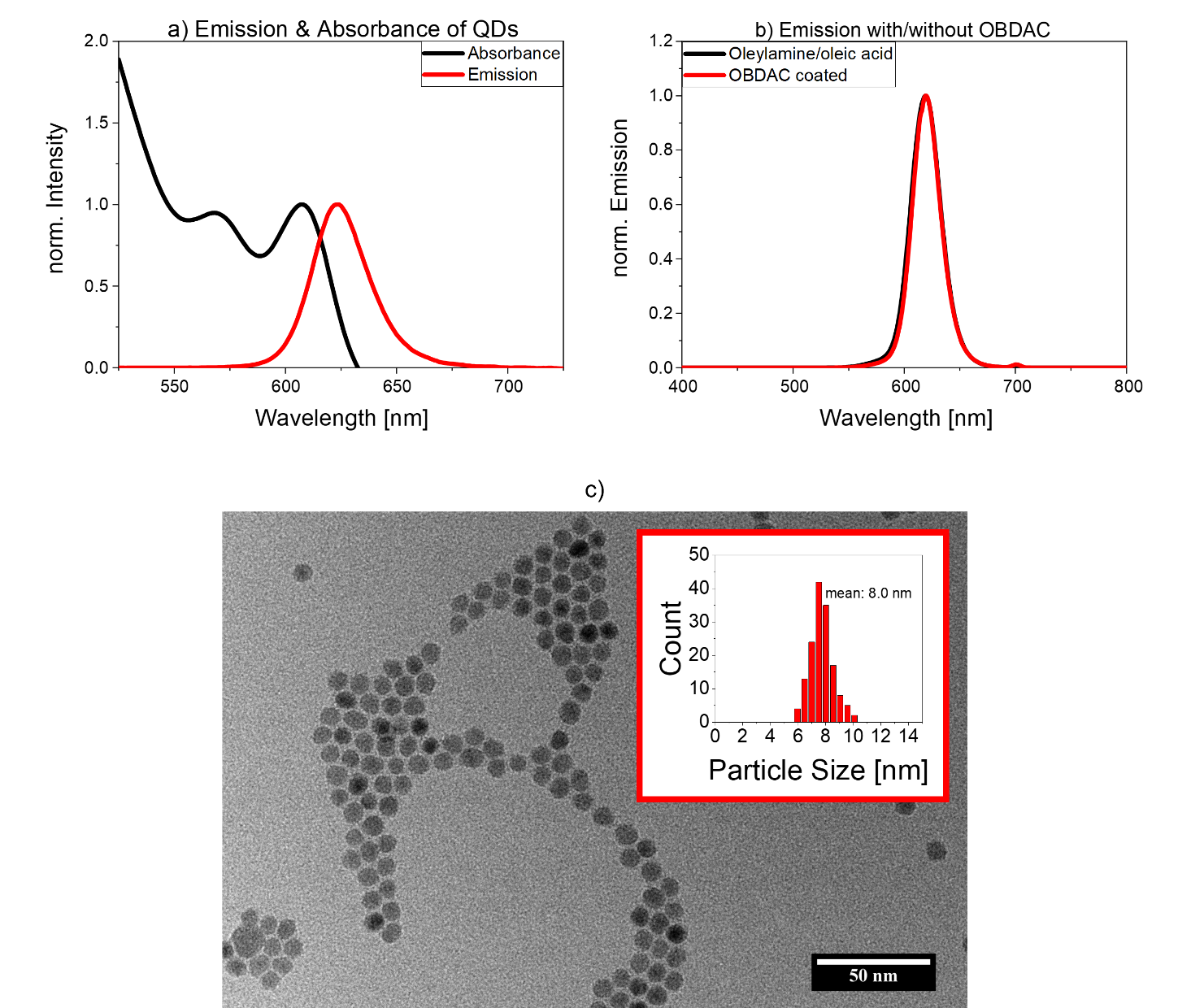


**Figure S2:** a) Emission (λ_exc_ = 350 nm) and absorbance spectra of OA/OLA-stabilized CdSe/CdS semiconductor quantum dots (QDs) in toluene, b) Emission spectra (λ_exc_ = 350 nm) of as-prepared CdSe/CdS-QDs with OA/OLA ligands and OBDAC-coated, OA/OLA-stabilized CdSe/CdS-QDs, both in toluene and c) TEM image of OA/OLA-stabilized CdSe/CdS-core/shell-QDs with size distribution.

The Cd concentration of the CdSe/CdS semiconductor quantum dot (QD) dispersion was determined to be 129 mmol/L by atomic absorption spectroscopy (AAS), the mean size of the particles was calculated from the TEM images to 8.0 nm (standard deviation 0.8 nm). For the OBDAC-coated, OA/OLA-stabilized QDs, the photoluminescence quantum yield (PLQY) decreased slightly to 68 % compared to the initial OA/OLA stabilized QDs with a value of 72 %.

1. **Photoluminescence properties of Nile Red- and QD-encoded PS microbeads**

**Table S3**: Summary of the PL properties of Nile Red (NR), CdSe/CdS-QDs and the resulting encoded PS microbeads.

| **Sample** | **Emission Maximum / nm** | **FWHM / nm** | **PLQY / %** |
| --- | --- | --- | --- |
| NR in styrene | 599 | 68.7 | 86 |
| NR-encoded beads, crosslinked | 610 | 89.0 | 74 |
| NR-encoded beads, not crosslinked | 606 | 91.6 | 74 |
| OA/OLA stabilized QDs in toluene | 619 | 32.0 | 72 |
| OBDAC coated, OA/OLA stabilized QDs in toluene | 619 | 30.4 | 68 |
| Microbeads encoded with OA/OLA stabilized QDs, crosslinked | 622 | 28.6 | 29 |
| Microbeads encoded with OBDAC coated, OA/OLA stabilized QDs, not crosslinked | 622 | 26.9 | 15 |
| Microbeads encoded with OBDAC coated, OA/OLA stabilized QDs, crosslinked (optimized) | 624 | 30.9 | 34 |

Table S3 shows that the spectral width or Full Width at Half Maximum (FWHM) values of the optimized, QD-encoded bead emission band are very similar to those of the initial QDs. This indicates that QD size and size distribution did not change during the polymerization reaction.

1. **Absorbance spectra of polymer microbeads encoded with OBDAC-coated, OA/OLA-stabilized CdSe/CdS-QDs**


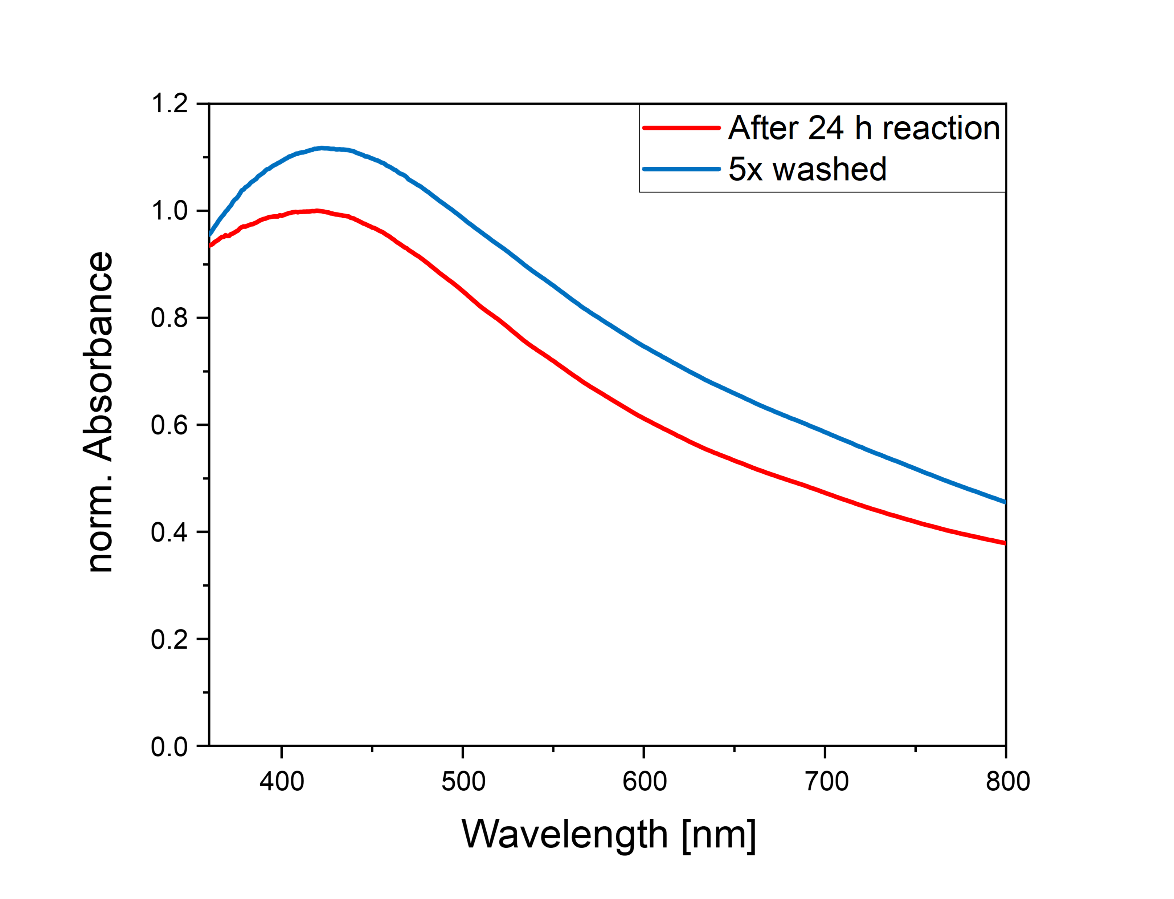


**Figure S4:** Absorbance spectra of DVB-crosslinked PS microbeads encoded with OBDAC-coated, OA/OLA-stabilized CdSe/CdS-QDs, directly and without purification after 24 h reaction time, and after five consecutive washing steps. Synthesis conditions: 70 °C, 100 mg AIBN, 36.6 mg PEG-b-PCL, 70 rpm stirring speed, 24 h reaction time.

The absorbance spectra of QD-encoded microbeads in Figure S4 show a higher absorbance for the washed beads (blue line) compared to those measured directly after the synthesis (red line). This can be attributed to an increase in the concentration of the bead solution caused by the loss of solvent (ethanol) during the washing steps.

1. **EDXS mapping of QD-encoded PS microbeads**


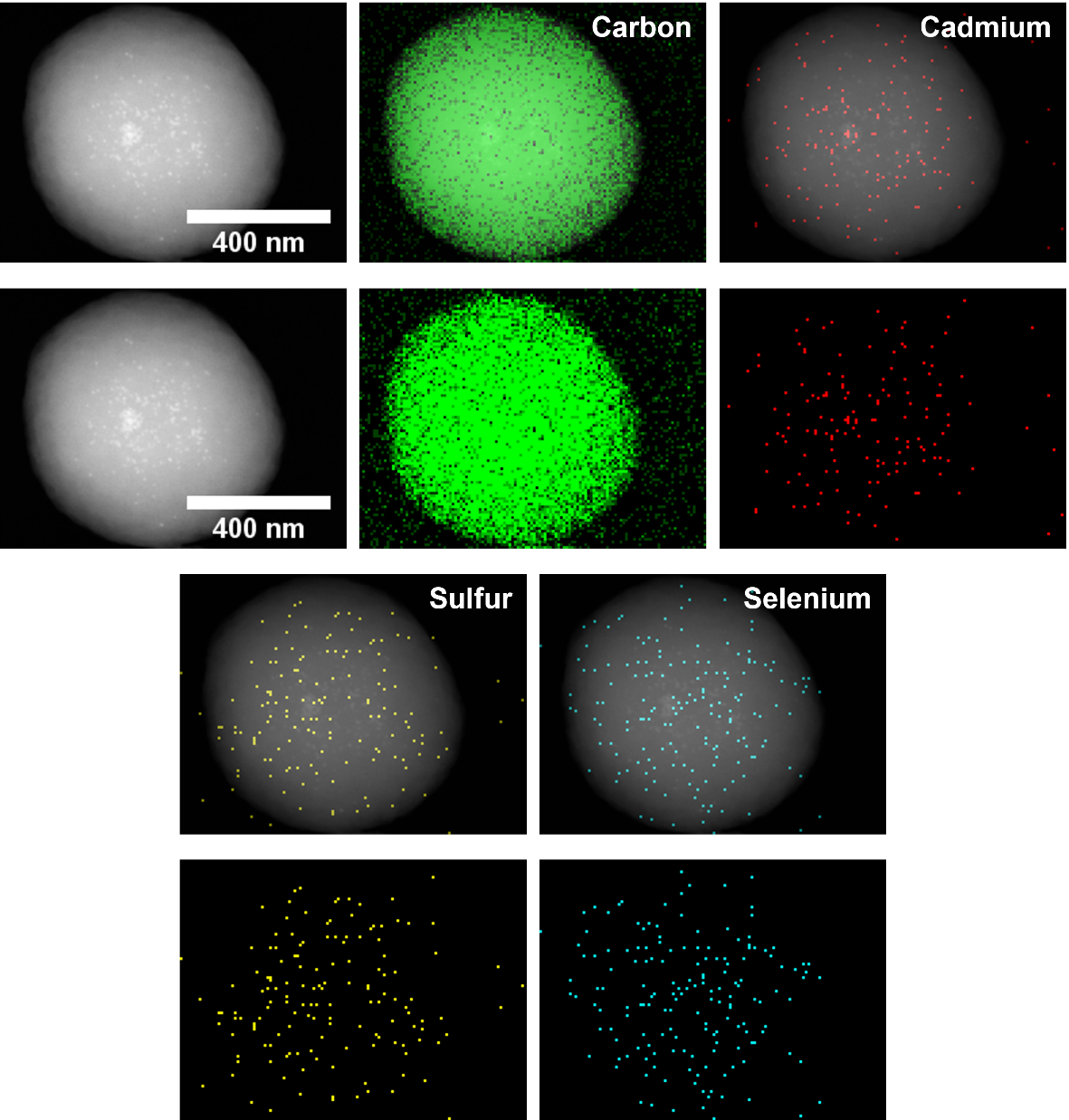


**Figure S5:** EDXS maps for different elements contained in a DVB crosslinked microbead encoded with OBDAC-coated, OA/OLA-stabilized CdSe/CdS-QDs. Reaction conditions for microbeads: 75 °C, 36.6 mg AIBN, 36.6 mg PEG-b-PCL, 70 rpm stirring speed, 24 h reaction time.

The EDXS maps of the different elements that are part of the OBDAC-coated, OA/OLA-stabilized CdSe/CdS-QDs show a clear location inside of the microbead. This confirms the presence of the QDs in the beads.

1. **Stability studies with NR- and QD-encoded PS microbeads**


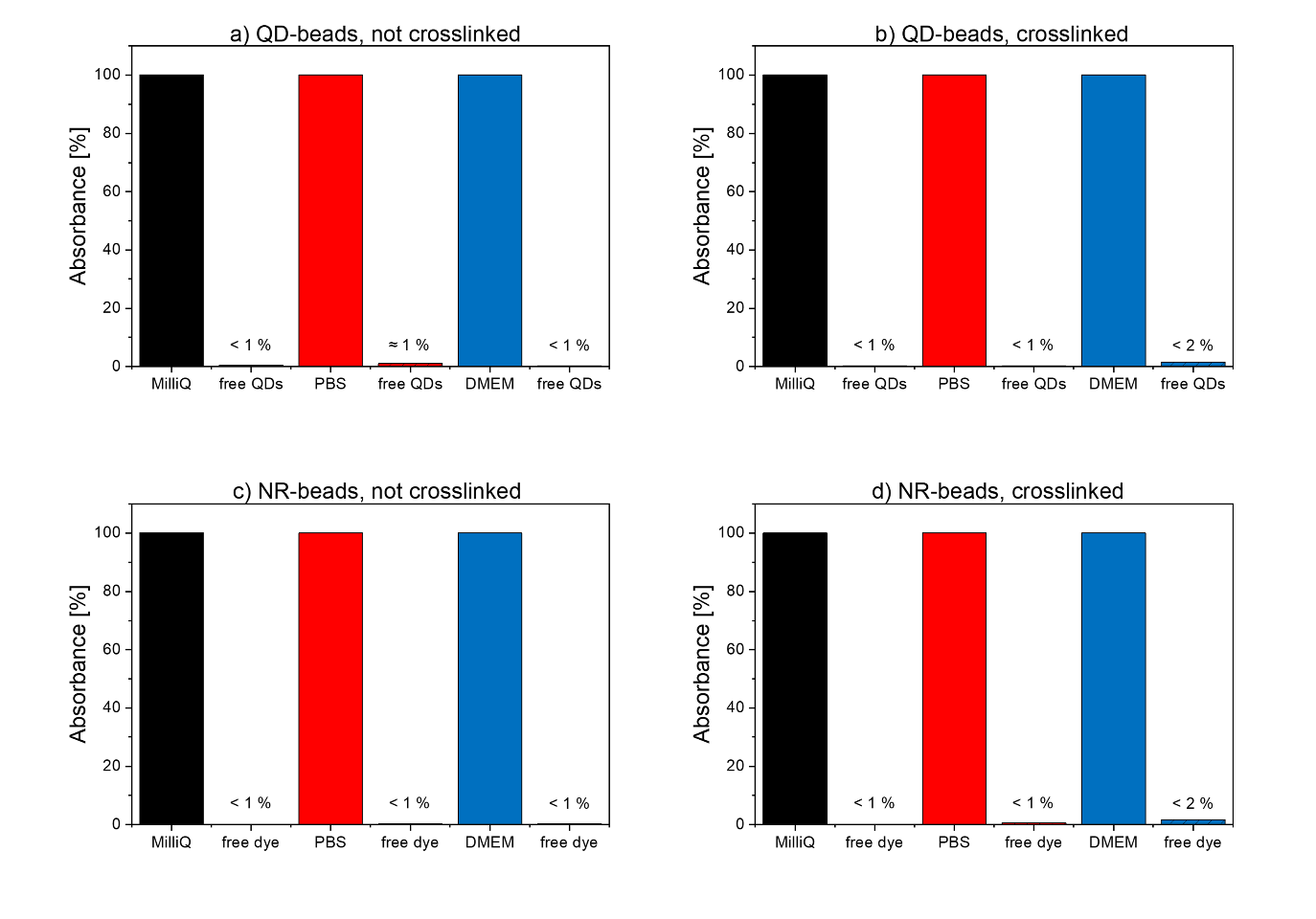


**Figure S6:** Comparison of the absorbance of purified, encoded microbeads incubated in different media (MilliQ water, phosphate-buffered saline solution (PBS), Dulbecco′s Modified Eagle′s Medium (DMEM)) for one hour at 37 °C and the respective supernatants after centrifugation at 12000 rcf for 10 min (Eppendorf Microcentrifuge 5415 D) with centrifugal filter units (10 kDa, Amicon Ultra, Merck Millipore) for a) microbeads without crosslinking, encoded with OBDAC-coated, OA/OLA-stabilized CdSe/CdS-QDs; b) DVB-crosslinked microbeads encoded with OBDAC-coated, OA/OLA-stabilized CdSe/CdS-QDs; c) microbeads without crosslinker, encoded with NR, and d) DVB crosslinked microbeads encoded with NR.

1. **Photostability of NR- and QD-encoded PS microbeads**


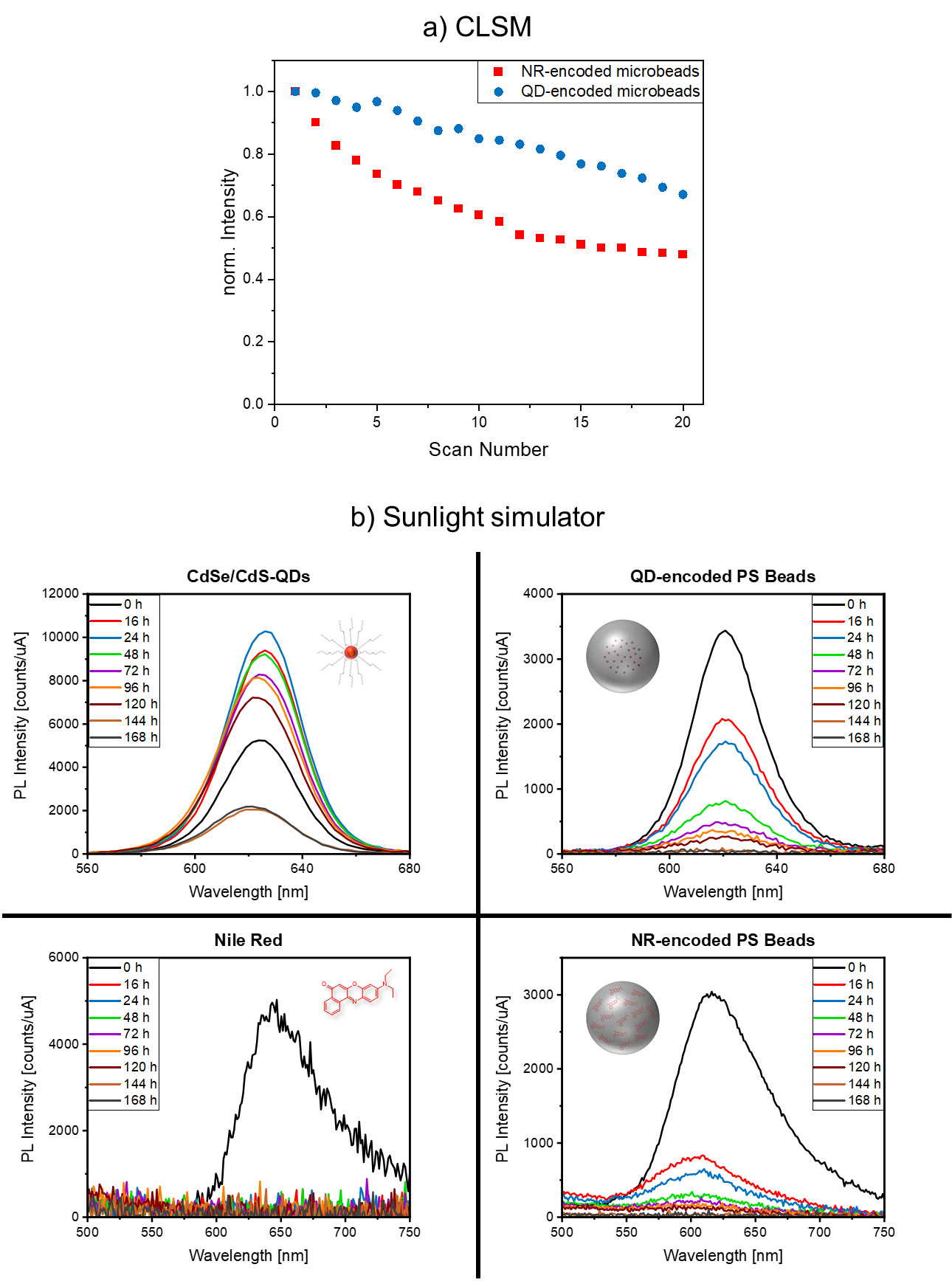


**Figure S7:** Short-term and long-term photostability studies of NR- and QD-encoded PS microbeads, a) Short-term light exposure utilizing a CLSM (time per scan 2.1 s, 1 mW excitation power in the beam path) and b) long-term exposure with a sunlight simulator (650 W/m^2^ illumination), here in comparison to the dye NR and the initial QDs. In the case of the CLSM measurements, the luminescence intensities and their changes were calculated by integrating the intensity of the region of interest (ROI) and normalizing the intensities by setting the start values to one. The spectra recorded after exposure with the sunlight simulator were also normalized by setting the start value to one.

As can be seen in Figure S7, the luminescence intensity of the QDs illuminated with the sunlight simulator initially increased (photobrightening) and then started to decrease after 24 h. We attribute the initial photobrightening to a healing of defects on the QD surface. In contrast, the luminescence of the QD-encoded beads did not undergo photobrightening yet revealed a slow decrease in luminescence. In the case of the bead-incorporated QDs, surface defect healing apparently occurred already during microbead synthesis. The light-induced blue shift in QD luminescence is ascribed to a decrease in QD size. In all cases, the QD systems revealed an improved phtostability compared to the dye systems.
